# Molecular Detection and Genotyping of Intestinal Microsporidia from Stray Dogs in Iran

**Published:** 2019

**Authors:** Moein DELROBAEI, Shahram JAMSHIDI, Parviz SHAYAN, Elahe EBRAHIMZADE, Iraj ASHRAFI TAMAI, Mostafa REZAEIAN, Hamed MIRJALALI

**Affiliations:** 1. Department of Internal Medicine, School of Veterinary Medicine, University of Tehran, Tehran, Iran; 2. Department of Parasitology, School of Veterinary Medicine, University of Tehran, Tehran, Iran; 3. Department of Microbiology and Immunology, School of Veterinary Medicine, University of Tehran, Tehran, Iran; 4. Department of Medical Parasitology and Mycology, School of Public Health, Tehran University of Medical Sciences, Tehran, Iran; 5. Foodborne and Waterborne Diseases Research Center, Research Institute for Gastroenterology and Liver Diseases, Shahid Beheshti University of Medical Sciences, Tehran, Iran

**Keywords:** Stray dogs, Microsporidia, *Enterocytozoon bieneusi*, Genotyping, Iran

## Abstract

**Background:**

Microsporidia as one of the most important pathogens in veterinary and agricultural settings, have emerged in immunocompromised patients in Iran. To date, different *Enterocytozoon bieneusi* genotypes have been identified in humans and animals, supporting the possibility of zoonotic zoonosis transmission potential. The aim of this study was to evaluate the distribution of *E. bieneusi* genotypes among overpopulated stray dogs in vicinity of Tehran, the capital city of Iran.

**Methods:**

Totally, 75 stool and 75 urine samples were obtained from 75 stray dogs during the time period from Mar 2015 to Oct 2015. DNA extraction was performed on all the samples and specific fragment of small subunit ribosomal RNA gene of *E. bieneusi* and *Encephalitozoon* spp. was amplified. Furthermore, specific primers targeting the internal transcribed spacer region of *E. bieneusi* were applied to determine the genotype of the microorganism.

**Results:**

Microsporidia was detected in 5.3% of stool samples, while none of the urine samples was positive for microsporidia species. Overall, 440 bp fragment of *E. bieneusi* was amplified in all the samples and there was no amplification for *Encephalitozoon* spp. The results of sequencing of 410 bp fragment of internal transcribed spacer region showed that all the *E. bieneusi* were genotype D.

**Conclusion:**

*E. bieneusi* was the most prevalent microsporidian species in the stray dogs and all the positive isolates were characterized as genotype D.

## Introduction

Microsporidia species are obligate intracellular spore-forming single-celled microorganisms. These organisms are ubiquitous in nature as well as infecting both invertebrate and vertebrate animals. More than 1200 species of the phylum Microsporidia have been recently reclassified as fungi. Until now, more than 1200 species of the phylum have been classified into approximately 100 genera (1–3). The size of microsporidian spores varies from approximately 1to 10 μm with a robust and rigid wall to protect the organisms from environmentally damaging conditions (4, 5).

*Enterocytozoon bieneusi* and *Encephalitozoon* species including *E. cuniculi*, *E. intestinalis,* and *E. hellem* are the major species of microsporidia, leading gastrointestinal disorders in humans and wide range of animals (6–8). *E. bieneusi* is the most commonly identified microsporidia in human subjects firstly identified in a HIV/AIDS patient (9). Thereafter, *E. bieneusi* has been introduced from pigs (6), and several wild and domestic animals (10). Despite epidemiological studies on *E. bieneusi* infection in humans and animals, the main route of transmission and reservoirs remain ambiguous. However, most of the infections result from fecal-oral transmission of spores from infected humans and animals through contaminated food, water, and even inhalation. There is evidence showing water resources as suitable environment for spores to survive and transmit to a new host (11, 12).

Genotyping and molecular analysis of *E. bieneusi* considered as an accurate tool to clarify some transmission aspects. Differences among *E. bieneusi* genotypes are not morphologically traceable; therefore molecular methods should be applied to characterize genotypes of the parasite. According to nucleotide diversity through Internal Transcribed Spacer (ITS) region of Small Subunit rRNA (SSU rRNA) gene, 201 genotypes have been identified so far. Meanwhile, about 54 genotypes have been reported from only human, 33 genotypes from both humans and animals and 102 genotypes from only animals suggesting zoonotic and/or anthroponotic nature of certain genotypes (10, 13). However, distribution and the source of infection of the different genotypes vary in different geographical regions (14, 15). In Iran, *E. bieneusi* have been reported from pet, farm and laboratory animals, pigeons, HIV+/AIDS patients, transplant recipients and cancer patients implicating animals as potential sources of microsporidia infection for humans (16–21).

In the current study, overpopulated stray dogs in vicinity of Tehran, Iran by having free access to the surface water as well as agricultural setting can distribute the microsporidia spores through the environment and may play a significant role in the epidemiology of microsporidia.

The aim of this study was evaluation of the existence and distribution of microsporidian species and genotypes in the urine and feces of stray dogs by molecular methods.

## Materials and Methods

### Sampling

Overall, 75 stool samples and 75 urine samples were obtained simultaneously from 75 stray dogs in the vicinity of Tehran Province, Iran between Mar 2015 and Oct 2015. All dogs were apparently healthy without any clinical abnormalities and returned to the environment after the end of sampling. Fecal samples were collected from the rectum by using a sterile spatula and placed in sterile container. Urine samples were collected with sterile syringes directly from urinary bladder and stored in sterile container. All the samples transported to the laboratory on ice and stored at −20 °C until molecular analysis.

This study has been approved by the Iranian Laboratory Animal Ethics Committee under the supervision of the Iranian Society for the Prevention of Cruelty to Animals.

### DNA extraction and Nested PCR

Urine samples were centrifuged at 10000 ×g for 5 min, the supernatant was discarded and then urine sediment together with stool samples were introduced to commercially available QIAamp DNA Mini Kit and QIAamp Stool Mini Kit (Qiagen, Hilden, Germany), respectively. DNA extraction was done according to the manufacturer’s instructions and finally purified DNA was stored at −20 °C until use.

Nested PCR was performed using previously introduced genus specific primers with some modifications in EncepR (20) to identify the existence of microsporidia spores. Briefly, expected 779 bp fragment of SSU rRNA gene of *Encephalitozoon* spp. and *E. bieneusi* was amplified by PMicF (5′-GGTTGATTCTGCCTGACG-3′) and PMicR (5′- CTTGCGAGCGTACTATCC - 3′). Thereafter, EnbF (5′- GGTAATTTGGTCTCTGTGTG - 3′), EnbR (5′- CTACACTCCCTATCCGTTC -3′) and EncepF (5′- AGTACGATGATTTGGTTG- 3′), EncepR (5′- ACAACACTATATAGTCCCGTC- 3′) were employed to amplify 440 bp and ≃ 630 bp fragments of *E. bieneusi* and *Encephalitozoon* spp., respectively. Another nested PCR was carried out using a set of specific primers targeting ITS fragment of *E. bieneusi* (22). Two tubes consisted of primers, PCR master mix and without template were used as negative control in each set of nested PCR. Subsequently, expected 410-bp fragment of ITS gene was sequenced using ABI sequencer to characterize genotypes of the positive samples.

#### Phylogenetic analysis

Phylogenetic tree of sequenced ITS fragment was constructed using Maximum-likelihood algorithm and tamura-3-parameter model in MEGA6 software. Bootstrapping (1000 replicates) statistically support the topology of tree. A number of sequences of previous studies on both human subjects and animals were also included together with our sequence to evaluate molecular distance between isolates.

## Results

**Fig. 1:**
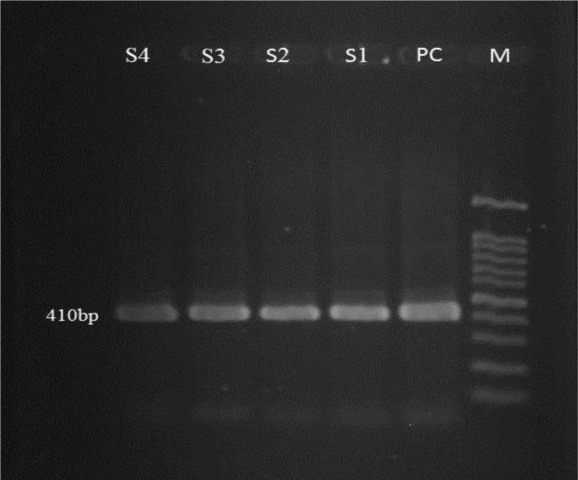
Gel electrophoresis of 410 bp targeting the ITS fragment of *E. bieneusi* using Nested-PCR, M: 100bp marker, S1 to S4: samples of *E. bieneusi*, PC: positive control

The nested PCR method with specific primers proved the presence of *Enterocytozoon* spores in 4 fecal samples of 75 stray dogs (5.3%). *Encephalitozoon* spp. were not found in the samples. For all molecularly positive samples, 410 bp fragment of ITS gene were amplified (Fig. 1).

None of the urine samples were positive. All the sequences were manually trimmed and edited and then compared with those sequences previously deposited in the GenBank database. Subsequently, all the positive samples were identified as genotype D and were submitted in GenBank (Accession No: KY066465, KY249119, KY249120, KY249121). Coinfection with different genotypes was not observed in the samples (Table 1). Phylogenetic analysis showed that all the genotypes D isolated in the current study were grouped together with other genotypes D, previously obtained from human and animal subjects.

**Table 1:** Source of spores, genotypes and accession numbers of *E. bieneusi* isolated from stray dogs

***No.***	***Specimen***	***Genotype***	***Accession no***
1	Stool	D	KY066465
2	Stool	D	KY249119
3	Stool	D	KY249120
4	Stool	D	KY249121

## Discussion

Microsporidia are emerging pathogens infecting almost all animal phyla. *E. bieneusi* is known as the most common microsporidian species identified in humans. Although the main source of microsporidia infection is still uncertain, some genotypes infecting humans have been identified in domestic and wild animals as well, which supports the hypothesis discussing possibility of zoonotic transmission (23). To our knowledge, this study is the first report that representing genotype D in stray dogs in Iran.

In the current study, the results obtained from nested PCR revealed that *E. bieneusi* was the only microsporidian species obtained from 5.3% of fecal samples collected from stray dogs. This result is in agreement with several studies have been performed in human and different animals, suggesting *E. bieneusi* as the predominant species of microsporidia. In Iran, of 142 stool samples collected from the animals with close contact to human, *E. bieneusi* identified in 3/30 (10%) of sheep, 2/39 (5.12%) of dairy cattle, 1/10 (10%) of rabbit, 3/26 (11.53%) of cats and 2/17 (11.76) ownership dogs, showing *E. bieneusi* as the most prevalent microsporidia (21). This species was also the most common species recognized in 13 out of 147 (8.8%) pigeons from various regions of Tehran, Iran (17). Even if the predominant species was *En. Cuniculi (*18 out of 100 dogs*), E. bieneusi* was identified in 8 out of 100 household dogs and 3 out of 40 cats (16).

Additionally, several studies have done in different countries indicating the predominance of this species all over the world; *E. bieneusi* was identified in 3 out of 36 dogs in Switzerland (24), 18/120 dogs in Colombia (25), 7/17 dogs in Spain (26), 2/26 dogs in China (27) and 2/79 dogs in Japan (28). However, *E. bieneusi* is known as the predominant microsporidia reported from human as well (29.30). *E. bieneusi* has been frequently identified in humans in Iran and has introduced as the most common microsporidian species. *E. bieneusi* in were identified 25/81 (30.86%) of HIV+/AIDS patients (20). Diarrhea caused by *E. bieneusi* was described in 3/44 (6.81%) liver transplant children (19).

Until now, 201 *E. bieneusi* genotypes have been reported worldwide. Among 102 *E. bieneusi* genotypes reported only in animals, genotypes PtEb IX, CHN5 and CHN6 considered as dog-specific genotypes identified only in dogs (13) and among 33 genotypes reported from humans and animals, genotypes D, WL11(Peru5), Peru6 and Type IV(Peru2) have been identified in both humans and dogs (10,13). *E. bieneusi* genotypes Peru 5 and K, previously reported as human pathogens, were identified for the first time in dog in Colombia (25). Furthermore, *E. bieneusi* genotype Peru 6 was identified in a dog in Portugal (31), a genotype previously reported only in humans, bird and cattle, suggesting a broad host adaptation of this parasite (13). Likewise, transmission of *E. bieneusi* between a child and Guinea pigs supports this hypothesis that even unique genotypes might have zoonotic potential (32). In the current study, all the positive samples were identified as *E. bieneusi* genotype D which is in agreement with other studies have been performed in Iran and different parts of the world introducing genotype D as the most prevalent genotype of *E. bieneusi*. Genotype D was the most prevalent genotype (46.2%) in urban pigeons in Tehran, Iran (17). Genotyping has not been performed on the other isolated *E. bieneusi* from animals in Iran.

Furthermore, genotype D has been reported as the most common genotype in humans in Iran. This genotype has been isolated in liver transplant children (19) and HIV+/AIDS patients (18). Genotype D has been identified as the most frequent genotype in immunocompromised patients (22). However, genotype D is known as the most prevalent genotype that is not host-specific and has been isolated from human, pig, wild boar, cattle, beaver, fox, muskrat, raccoon, river otter, falcon, pigeon, horse, dog, mice, baboon, rhesus macaque, cynomolgus monkey and white-headed langur, almost all over the world (10, 13). In the current study, all of the isolated *E. bieneusi* characterized as genotype D. Since the excretion of spores through the environment considered being intermittent, the true prevalence of the parasite might be higher (33).

**Fig. 2:**
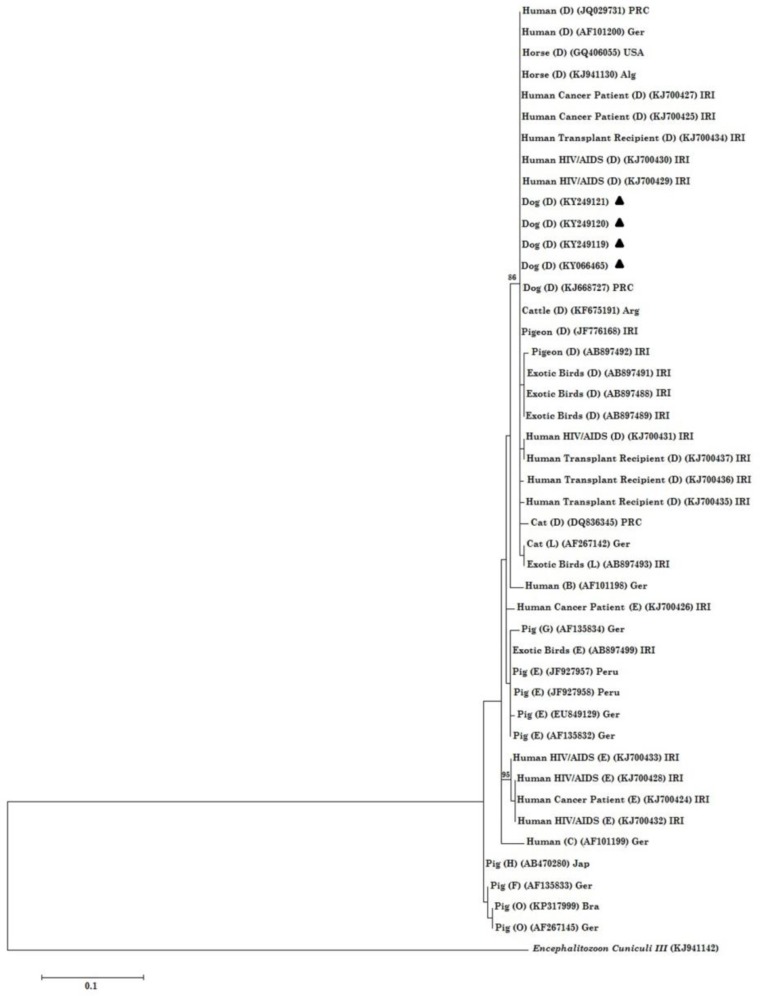
Phylogenetic analysis of ITS nucleotide sequences of *E. bieneusi* isolates recovered from stray dogs in Iran. The tree was constructed using the Maximum Likelihood test and the Tamura 3-parametermodel as implemented in theMEGA6 software. *En. cuniculi* genotype III was used as anoutgroup. Bootstrap under 75 were deleted. Abbreviations: Ger, Germany; Jap, Japan; Bra, Brazil; IRI, Islamic Republic of Iran; Arg, Argentina; PRC, People’s Republic of China; Alg, Algeria; USA, United State of America All the sequences retrieved from the current study are characterized with black-filled triangles

Finally, according to the phylogenetic tree (Fig. 2), all the sequences generated in the current study were placed in a branch together with other sequences introduced from human, dog, cattle, pigeon, and horse. This finding is in agreement with the study that showed there was no relationship between genotype and type of immunodeficiency and host (22). The results of the phylogenetic analysis also represented that *E. bieneusi* isolated from both human and animal subjects were placed beside each other stating high possibility of zoonotic transmission of this microorganism.

Accordingly, in the current study the isolation of *E. bieneusi* genotype D in stray dogs isolated from humans either, not only as a sole prerequisite for demonstrating the zoonotic potential of *E. bieneusi,* but there is no evidence of a transmission barrier between human and stray dogs and therefore, stray dogs could play an important role in epidemiology of microsporidia infection.

## Conclusion

Public health concerns of overpopulated stray dogs around the cities with free access to water resources, agricultural farms as well as close contact with humans must be considered not only as a source of the re-emerging zoonoses like rabies, hydatidosis, and leishmaniasis but also emerging pathogens like microsporidia. However, more studies should be performed about the distribution of microsporidia genotypes in different water sources in Iran, where microsporidiosis has been reported in human and animals.
